# Seasonal adherence to, and effectiveness of, subcutaneous interferon β-1a administered by RebiSmart® in patients with relapsing multiple sclerosis: results of the 1-year, observational GEPAT-SMART study

**DOI:** 10.1186/s12883-018-1179-0

**Published:** 2018-11-06

**Authors:** Spyros N. Deftereos, Evangelos Koutlas, Efrosini Koutsouraki, Athanassios Kyritsis, Panagiotis Papathanassopoulos, Nikolaos Fakas, Vaia Tsimourtou, Nikolaos Vlaikidis, Antonios Tavernarakis, Konstantinos Voumvourakis, Michalis Arvanitis, Dimitrios Sakellariou, Filippo DeLorenzo

**Affiliations:** 1 Merck Hellas, 41-45 Kifisias av, 15123 Athens, Greece; 2 Neurology Department, Papageorgiou Hospital, Thessaloniki, Greece; 3 Neurology Department, Aristotle University of Thessaloniki, Thessaloniki, Greece; 4 University of Ioannina Neurology Department, Ioannina, Greece; 5 Neurology Department, University of Patras, Patras, Greece; 6 Neurology Department, 401 Army Hospital of Athens, Athens, Greece; 7 Neurology Department, University of Thessaly, Larissa, Greece; 8 Neurology Department, Evangelismos Hospital, Athens, Greece; 9 B Neurology Department, University of Athens, Athens, Greece; 10 Private Practice, Athens, Greece

**Keywords:** Multiple sclerosis, Interferons, Rebif, Rebismart, Treatment adherence and compliance, Clinical efficacy

## Abstract

**Background:**

Little is known about whether tolerability and adherence to treatment can be influenced by weather and temperature conditions. The objective of this study was to assess monthly and seasonal adherence to and safety of sc IFN-β1a (Rebif®, Merck) in relapsing-remitting multiple sclerosis (RRMS) patients using the RebiSmart® electronic autoinjector.

**Methods:**

A multicentre, prospective observational study in Greece in adult RRMS patients with EDSS < 6, under Rebif®/RebiSmart® treatment for ≤6 weeks before enrollment. The primary endpoint was monthly, seasonal and annual adherence over 12 months (defined in text). Secondary endpoints included number of relapses, disability, adverse events.

**Results:**

Sixty four patients enrolled and 47 completed all study visits (Per Protocol Set - PPS). Mean annual adherence was 97.93% ± 5.704 with no significant monthly or seasonal variations. Mean relapses in the pre- and post- treatment 12-months were 1.1 ± 0.47 and 0.2 ± 0.54 (*p* < 0.0001, PPS). 10 patients (22%) showed 3-month disability progression, 19 (40%) stabilization and 18 (38%) improvement. EDSS was not correlated to pre- (*r* = 0.024, *p* = 0.87) or post-treatment relapses (*r* = 0.022, *p* = 0.88).

**Conclusion:**

High adherence with no significant seasonal or weather variation was observed over 12 months. While the efficacy on relapses was consistent with published studies, we could not identify a relationship between relapses and disability.

**Trial registration:**

Greek registry of non-interventional clinical trials ID: 200136, date of registration: February 18th, 2013.

## Background

Adherence to treatment in Multiple Sclerosis (MS) is an important determinant of long-term outcomes, as suggested by the World Health Organization [1] and evidenced by several published studies [2–4]. However, the need for long-term treatment and the frequently debilitating nature of the disease make treatment adherence particularly challenging. This may impact disease progression, as on the one hand up 72% of patients do not adhere to disease-modifying MS treatments according to published studies [2, 5, 6], while on the other poor adherence has been associated with a higher rate of relapse [6].

The interferons (beta-1a and beta-1b) are among the first Disease Modifying Drugs (DMDs) that were approved for MS. These platform therapies are frequently associated with flu-like syndrome and injection-site reactions, which are among the reasons of non-adherence according to some studies [7]. Taking into account that the flu-like syndrome comprises a constellation of symptoms some of which may be more difficult to tolerate when the weather is hot, such as fever, chills and headache, we asked whether seasonal variation of weather conditions affects adherence to interferon treatment. As higher temperatures are typically observed in the Mediterranean countries, especially during the summer period, any effects of seasonal variation on adherence would be expected to be more pronounced in these countries. We, therefore, studied the seasonal variation of the adherence to sc IFN-β1a tiw (Rebif®), administered through the RebiSmart® autoinjector device, for a 12-month period, in patients with Relapsing-Remitting Multiple Sclerosis (RRMS) in Greece. Rebif® safety, including the occurrence of flu-like, was also studied.

## Methods

The GEPAT-SMART study (Greece Epidemiological Project on Adherence and Temperature Using RebiSMART®) was a multicentre, prospective, observational study carried out at 9 sites in Greece (Greek registry of non-interventional clinical trials id: 200136, date of registration: February 18th, 2013 [8]). The recruitment period lasted from February 2013 to February 2014. The last patient follow-up ended on April 2015. The study was carried out in accordance with the Declaration of Helsinki and applicable national regulatory requirements and was approved by local ethics committees at each study site [ethics committee of the Papageorgiou Hospital of Thessaloniki (reference number 161/20.9.2012), ethics committee of the AHEPA Hospital (reference number 32/5.12.2012), ethics committee of the University Hospital of Ioannina (reference number 754/12.11.2012), ethics committee of the University Hospital of Patras (reference number 83/7.2.2013), ethics committee of the 401 Army Hospital of Athens (reference number 15/2012), ethics committee of the University Hospital of Larissa (reference number 19/13.11.2012), ethics committee of the Papanikolaou Hospital of Thessaloniki (reference number 11/3.10.2012), ethics committee of the Evangelismos Hospital (reference number 345/13.12.12), ethics committee of the Attiko Hospital (reference number 10/5.10.2012)]. Patients were enrolled after written informed consent had been obtained.

### Participants

Inclusion criteria were 1) RRMS diagnosis (revised McDonald criteria (2010)), 2) Rebif® multi-dose injected by RebiSmart® prescribed according to the approved Summary of Product Characteristics (SmPC) within six (6) weeks prior to their enrolment into the study, 3) capable to handle RebiSmart®, 4) willing and capable to comply will all study requirements and procedures, 5) 18 to 65 years old and 6) Expanded Disability Scale Score (EDSS) < 6 at enrollment.

Exclusion criteria were 1) presence of any contraindication mentioned in the locally approved SmPC, 2) severe relapse within 30 days before study treatment commencement, 3) visual or physical impairment precluding them from self-injecting with RebiSmart®, 4) MS therapy within 6 months prior to study, 5) current or past (within the last 2 years prior to study enrolment) history of alcohol or drug abuse, 6) participation in another clinical trial during the last 30 days prior to study treatment commencement. Female subjects who were pregnant or breast-feeding were also excluded. Female patients with childbearing potential had to utilize a highly effective method of contraception for the duration of the study.

### Administration of the study drug

All patients were provided with a RebiSmart® device (Merck, Darmstadt, Germany) for self-administration of serum-free Rebif® 44 μg or 22 μg sc three times weekly (tiw) for 12 months or until early discontinuation (ED). RebiSmart® is a CE-certified medical device. The dose of Rebif® was titrated over the first 4 weeks in accordance with the drug labeling information; the final dose was at the discretion of the treating physician and based on the recommendations in the drug labeling information.

### Patient assessments

Following a pre-study evaluation visit, patients attended the study site at Study Day 1 (baseline), Month 6, and Month 12. At the baseline visit, all patients provided written informed consent and information on demographics, medical history, concomitant diseases, and MS history, including the number and characteristics of relapses in the past 12 months, was collected. At each post-baseline visit, the investigators recovered adherence data from the autoinjector. Reasons for missed injections were recorded in a patient diary. Relapse assessment, EDSS score, MS-related concomitant medication, vital signs, on-going therapy with Rebif® (including dose), and adverse events (AEs) were also recorded.

### Study endpoints

The primary endpoint was the monthly, seasonal and annual adherence rate over the 12-month study treatment. Adherence rate was defined as 100 × number of injections actually administered divided by the expected number of injections over the defined time period (month, season, year), as captured by RebiSmart®. Secondary endpoints were: 1) reasons for missed injections, 2) proportion of patients free of relapses at month 12, 3) mean number of relapses at 12 months, 4) proportion of patients without progression of 3-month confirmed disability, at 12 months. Disability progression was defined as worsening by at least 0.5 EDSS points from baseline, 5) proportion of patients who discontinued prematurely the study treatment and the reasons for discontinuation. All (Serious) Adverse Events [(S)AEs] and Adverse Drug Reactions [(S)ADRs] were also recorded, 6) Patient evaluation of RebiSmart® based on a Convenience Questionnaire.

The following criteria were to be met for establishing an MS relapse: 1) Neurological abnormality, either newly appearing or re-appearing, at least 30 days after the onset of a preceding clinical event, with > = 24 h duration, 2) absence of fever (temperature > 37.5C) or known infection and 3) objective neurological impairment, correlating with the patient’s reported symptoms, defined as either increase in at least one of the functional systems of the EDSS domain or increase of the total EDSS score. Severity of relapses was described as mild, moderate, or severe according to the Activities of Daily Living criteria [9]. AEs were classified according to MedDRA v14.0 [10].

### Sample size

The calculation of the sample size was based on the primary endpoint of the study. Due to the lack of literature data regarding the seasonal and monthly adherence, the adherence over the 12-month treatment period was used. According to available data the expected adherence during the study period was expected to be approximately 70% and the standard deviation 15% [11]. Therefore, 70 patients would be required to estimate the mean adherence rate with accuracy of less than ± 4%.

### Statistical analyses

This manuscript was development according to the STROBE (STrengthening the Reporting of OBservational studies in Epidemiology) guideline for reporting observational studies [12]. Descriptive statistics were calculated for all study variables. Summary statistics for categorical variables were presented as the number and percent of subjects in each category.

Seasonal and monthly variance of the adherence level was analyzed by One Way Analysis of Variance (ANOVA). Pre- and post-treatment relapse rate was compared by the Wilcoxon signed-rank test. Pearson’s r was used to study correlation between variables. The level of significance was set to 5% (two-sided). Descriptive statistics were used for AEs and SAEs. Adverse events where handled according to the study protocol.

Statistical analyses sets were performed in the following sets:

- Full analysis set (FAS): all recruited subjects who fulfilled the inclusion/exclusion criteria.
- Per-protocol set (PPS): all FAS subjects who completed all study visits.
- Safety set: all study patients who actually received at least one dose of treatment for MS following informed consent.

No replacement policy existed in this study for drop-out patients and missing data.

## Results

### Patient demographics

**Fig. 1:**
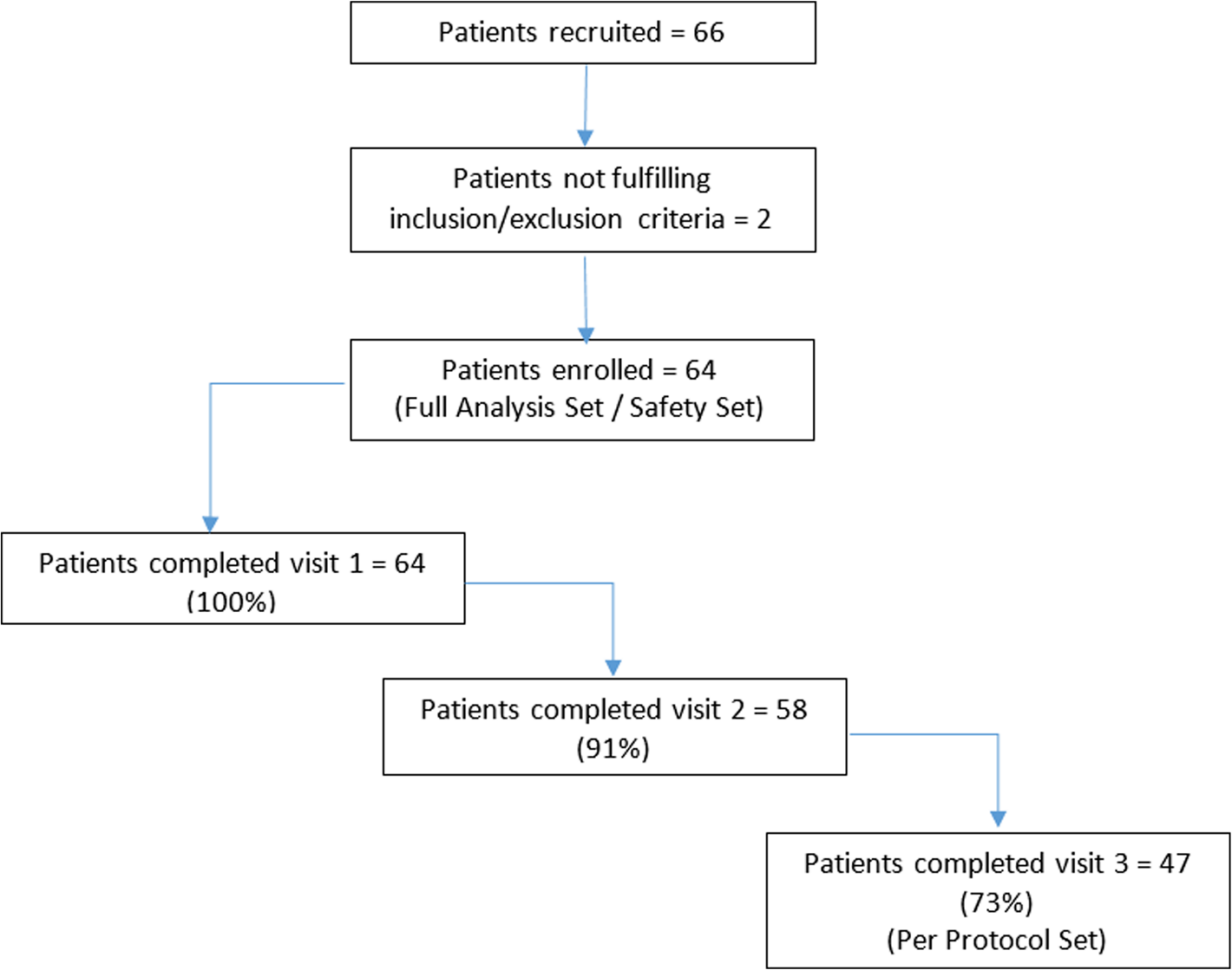
Patient Disposition

Sixty four of the 66 patients that started documentation received at least one dose of Rebif® and were included in the Safety Set and FAS, while the remaining two did not fulfill the inclusion/exclusion criteria and were not enrolled. Of these, 58 patients (87.9%) completed the month-6 visit, and 47 (71.2%) completed the month-12 visit. Patient disposition is shown in Fig. 1 and demographics are shown in Table 1. Baseline MS characteristics are shown in Table 2.

**Table 1:** Patient demographic characteristics (Full Analysis Set)

		n (%)
Gender	Males	14 (21.9)
	Females	50 (78.1)
Age (yrs)	n, mean ± sd	64, 36.2 ± 11.22
	min-max	18.3–68.8
Weight (kg)	n, mean ± sd	64, 69.9 ± 15.2
	min-max	47–127
Height (cm)	n, mean ± sd	64, 166.7± 9.24
	min-max	116–186
BMI (kg/m^2^)	n, mean ± sd	64, 25.16± 5.16
	min-max	18.40–46.2
Race	Caucasian	64 (100)
Place of Residence	Urban	46 (71.9)
	Semi urban	6 (9.4)
	Rural	12 (18.8)
Region	Attica	17 (26.6)
	Peloponnese	10 (15.6)
	Epirus	2 (3.1)
	Central Greece	2 (3.1)
	Central Macedonia	17 (26.6)
	Western Macedonia	7 (10.9)
	Eastern Macedonia / Thrace	1 (1.6)
	Crete	6 (9.4)
	Thessaly	2 (3.1)
	Ionian Islands	7 (10.9)
	North Aegean islands	0 (0.0)
	South Aegean Islands	0 (0.0)
Marital Status	Not married	29 (45.3)
	Married	32 (50)
	Widow/er	2 (3.1)
	Divorced	0 (0.0)
	Separated	1 (1.6)
Educational Status	0 yrs	0 (0.0)
	Elementary (1–6 yrs)	4 (6.3)
	High School/Lyceum (7–12 yrs)	32 (50.0)
	University (> 12 yrs)	28 (43.8)
Working status	Private Sector Employee	18 (28.1)
	Public Sector Employee	12 (18.8)
	Retired	2 (3.1)
	Free lancer	8 (12.5)
	Student	6 (9.4)
	Unemployed	18 (28.1)

**Table 2:** Summary of MS History (Full Analysis Set)

	n	mean ± sd	median	min-max
Years since MS diagnosis	64	2.1 ± 4.00	0.2	0.04–14.3
Mean number of relapses within the last 24 months prior to Rebif® Rebismart™ initiation	62	1.5 ± 0.76	1.0	0–4
Mean number of relapses in which corticosteroids were usedwere used were used	62	0.9 ± 0.71	1.0	0–3
Mean number of relapses within the last 12 months prior to Rebif® Rebismart™ initiation	63	1.1 ± 0.47	1.0	0–2
Mean number of relapses in which corticosteroids were used	62	0.9 ± 0.57	1.0	0–2

### Primary endpoint: 12-month and seasonal adherence

Mean adherence to Rebif®, administered through RebiSmart®, was 97.93% (±5.704) in FAS and 98.32% (±2.628) in PPS respectively (Table 3). No significant variations in monthly and seasonal adherence were noted (one-way ANOVA). Adherence did not vary significantly among different subgroups of the various demographic factors (Table 4).

**Table 3:** 12-month, seasonal and monthly adherence

	n	mean ± sd	median	min-max
12 month adherence to Rebif® - Rebismart® (Per Protocol Set)	46	98.32 ± 2.628	99.09	90.30–100
Study adherence to Rebif® - Rebismart® (Full Analysis Set)	62	97.93 ± 5.704	100	90.30–100
Seasonal adherence				
Jan-Mar	61	98.02 ± 6.879	100	57.97–100
Apr-Jun	57	98.36 ± 5.678	100	60.94–100
Jul-Sep	55	98.58 ± 3.276	100	81.63–100
Oct-Dec	56	97.91 ± 6.837	100	52.0–100
Monthly adherence				
Jan	60	97.54 ± 10.409	100	33.33–100
Feb	60	97.56 ± 8.513	100	54.55–100
March	59	98.34 ± 7.192	100	54.17–100
April	57	98.60 ± 6.826	100	50.00–100
May	57	98.67 ± 6.795	100	52.00–100
June	53	98.21 ± 5.560	100	65.00–100
July	52	98.45 ± 5.777	100	60.87–100
August	49	98.873 ± 2.935	100	86.67–100
September	52	98.46 ± 4.073	100	81.25–100
October	53	99.01 ± 2.963	100	86.67–100
November	52	97.933 ± 6.282	100	68.42–100
December	59	98.14 ± 6.721	100	52.00–100

*Annual adherence: 100 × (total no of injections in 12 months) / expected no of infections in the respective months*

*Seasonal adherence: 100 × (total no of injections in a 3 month-period) / expected no of infections during the same period*

*Monthly adherence: 100 × (total no of injections in specific month) / expected no of infections*

**Table 4:** Comparison of adherence in different subgroups, according to demographic factors

		12 months Compliance to			
		n	mean ± sd	min-max	*p*-value
Gender,	Males	13	98.55 ± 2.501	90.67–100	0.712
	Females	33	98.23 ± 2.708	90.30–100	
Age (yrs)	< 65	45	98.28 ± 2.645	90.30–100	NA
	≥ 65	1	100.00	100–100	
Race	Caucasian	46	98.32 ± 2.623	90.30–100	NA
	African	(−)			
	Asian	(−)			
	Other	(−)			
Place of Residence	Urban	33	98.42 ± 2.600	90.30–100	0.322
	Semi urban	4	99.66 ± 0.676	98.65–100	
	Rural	9	97.35 ± 3.105	90.67–100	
Region	Attica	10	98.05 ± 3.099	90.30–100	NA
	Peloponnese	6	97.89 ± 3.485	90.91--100	
	Epirus	2	98.81 ± 0.025	98.80–98-83	
	Central Greece	2	100 ± 0	100–100	
	Central Macedonia	14	98.68 ± 1.721	93.85–100	
	Western Macedonia	5	97.73 ± 4.041	90.67–100	
	Eastern Macedonia	(−)			
	Thrace	(−)			
	Crete	(−)			
	Thessaly	5	97.42 ± 3.108	92.09–100	
	Ionian Islands	2	100 ± 0	100–100	
	Northern/Southern Aeg. islands	(−)			
Marital Status	Unmarried	22	98.36 ± 2.914	90.30–100	0.988
	Married	22	98.26 ± 2.465	90.67–100	
	Widow/er	2	98.48 ± 2.143	96.97–100	
	Divorced	(−)			
	Separated	(−)			
Educational Status	0 yrs	(−)			
	Elementary (1–6 yrs)	4	96.64 ± 3.150	92.09–98.80	0.243
	High School/Lyceum (7–12 yrs)	22	98.08 ± 2.823	90.67–100	
	University (> 12 yrs)	20	98.92 ± 2.222	90.30–100	
Working status	Private sector employee	14	98.35 ± 2.619	90.30–100	0.993
	Public sector employee	9	98.73 ± 3.061	90.67–100	
	Retired	2	98.63 ± 0	98.63–98.63	
	Free lancer	5	98.12 ± 0.896	96.89–98.83	
	Student	4	98.46 ± 3.073	93.85–100	
	Unemployed	12	97.96 ± 3.185	90.91–100	

Thirty-one patients missed at least one dose of the study treatment. The main reasons for non-adherence were forgotten dose and other (12 subjects each, 18.8%), followed by presence of viral infection (flu, 15.6%) and absence from home (10.9%, Table 5).

**Table 5:** Reasons for non-adherence (Full Analysis Set)

	n (%)	Period of no injections	Events	Subject’s Location	Events
Subjects that missedat least oneinjection	31 (48.4)				
Reasons for missing the injections					
1. They forgot the injection	12 (18.8)	Week days	22	Home Area	25
		Bank Holidays	3	Out of Residence	2
		Holidays	2		
		Total Events	27		
2. They were not willing to inject for cosmetic reasons	0				
3. Absence from home	7 (10.9)	Week days	13	Home Area	11
		Bank Holidays	2	Out of Residence	6
		Holidays	3		
		Total Events	17		
4. Other reasons	12 (18.8)	Week days	55	Home Area	54
		Bank Holidays	2	Out of Residence	3
		Holidays	0		
		Total Events	57		
5. Pain reaction at injection site	0				
6. Flu-like illness	10 (15.6)	Week days	55	Home Area	53
		Bank Holidays	0	Out of Residence	2
		Holidays	0		
		Total Events	55		

### Secondary endpoints

#### Efficacy

**Fig. 2:**
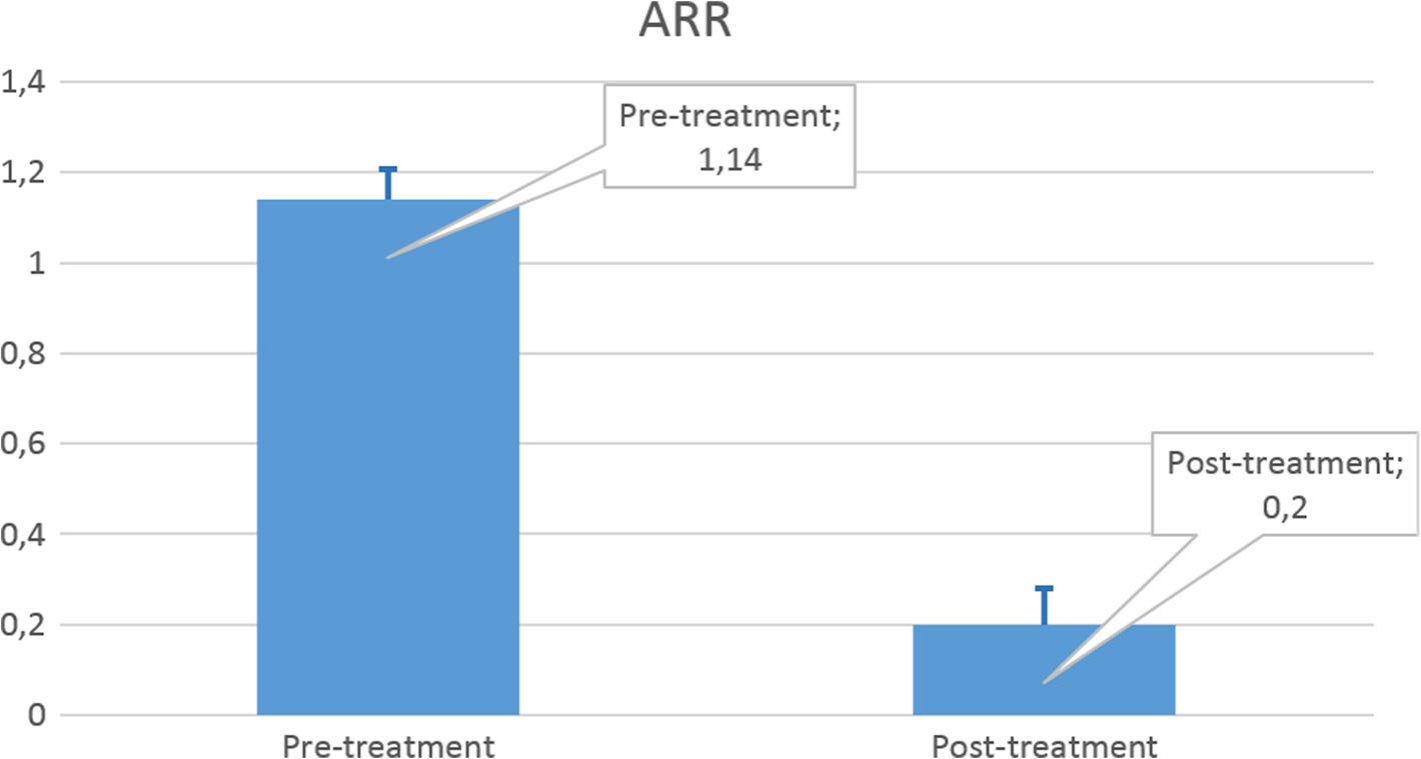
Mean number of relapses in the pre- and post-treatment 12 months

Among the 47 patients that completed all study visits (PPS), 6 (12.8%) relapsed with a mean number of relapses 1.3 ± 0.8 and 41 (87.2%) did not relapse. In the FAS 10 patients relapsed (15.6%) with a mean number of relapses 1.3 ± 0.6 and 54 (84.4%) did not relapse. Annual mean number of relapses in the PPS was 0.2 ± 0.54. This value was significantly lower compared to the mean number of relapses in the 12-month pre-Rebif® period (1.1 ± 0.47, *p* < 10^− 15^, Wilcoxon rank-sum test) (Fig. 2).

**Fig. 3:**
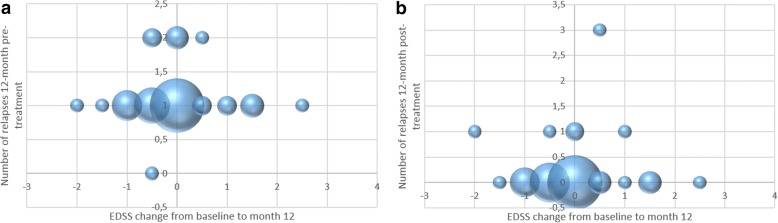
Change of EDSS between baseline and visit 3, at 12 months vs number of 12-month pre-treatment relapses (*n* = 47) (**a**) and 12-month post-treatment relapses (*n* = 47) (**b**). The size of the bubbles represents the number of observations at each point on the graph

**Fig. 4:**
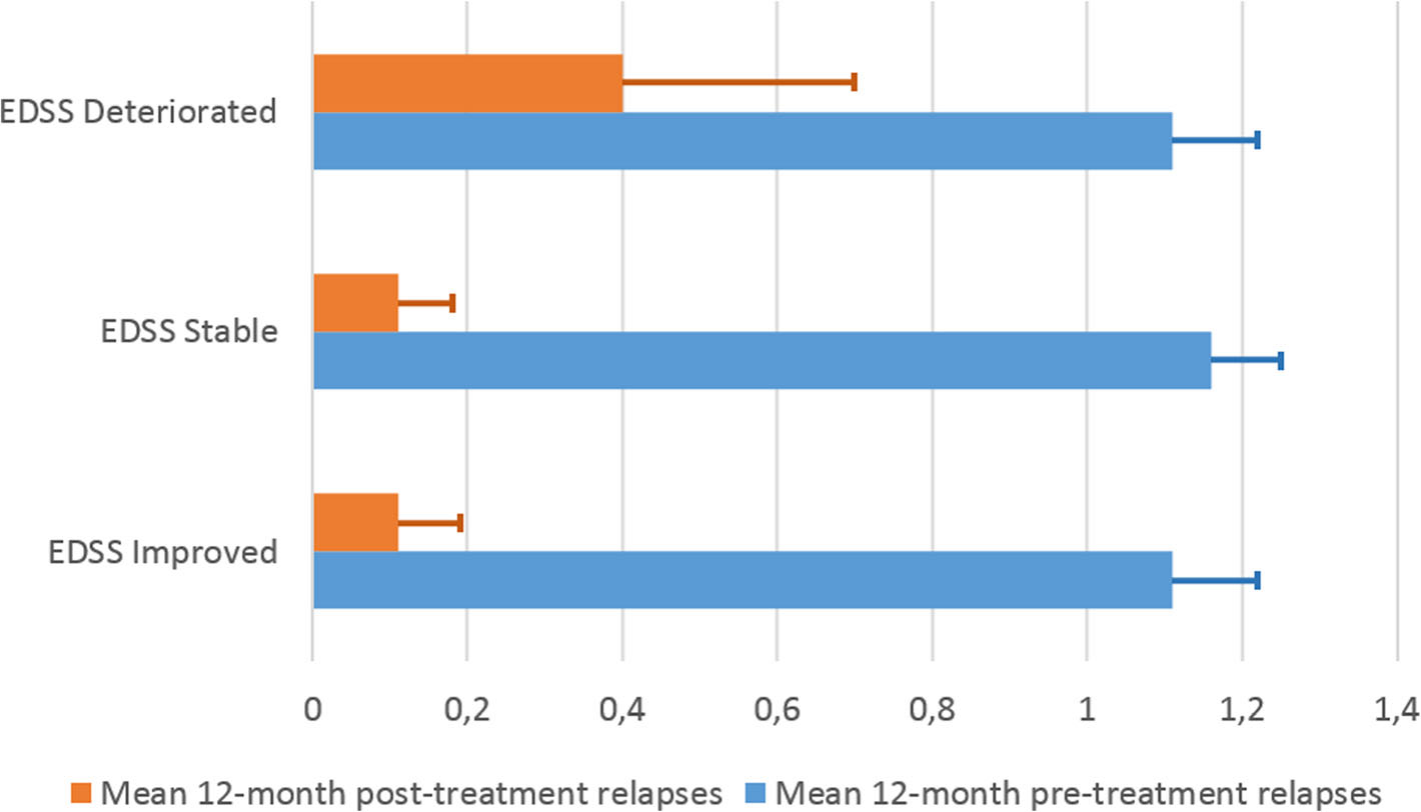
The mean number of relapses in the 12-month pre-treatment period did not differ significantly among patients in whom EDSS at the end of the trial was improved, stable or had worsened (one way anova, *p* = 0.94). Similarly, there were no statistically significant differences among these groups in the mean number of relapses in the 12-month post-treatment period (one way anova, *p* = 0.24). This result is in support of a dissociation between disability progression and relapses

3-month confirmed disability progression at the end of the study period was observed in 10 patients (21%), while in 19 (40%) patients EDSS remained stable and improved in 18 (39%). Median EDSS progression was 1 point (range 0.5–2.5) in the former group, while median improvement was 0.5 points (range 0.5–2) in the latter group. Overall, in the PPS mean EDSS change was not significantly different from zero with a mean of 0.17 ± 1.13 points (median 0). EDSS was related neither to the 12-month pre-treatment number of relapses (*r* = 0.024, *p* = 0.87), nor to the 12-month post-treatment number of relapses (*r* = 0.022, *p* = 0.88) (Fig. 3). Furthermore, mean 12-month relapses pre- and post-treatment were not significantly different among those in whom EDSS improved, remained stable or deteriorated (one-way ANOVA, *p* = 0.94 and 0.24 respectively, Fig. 4).

#### Safety

**Fig. 5:**
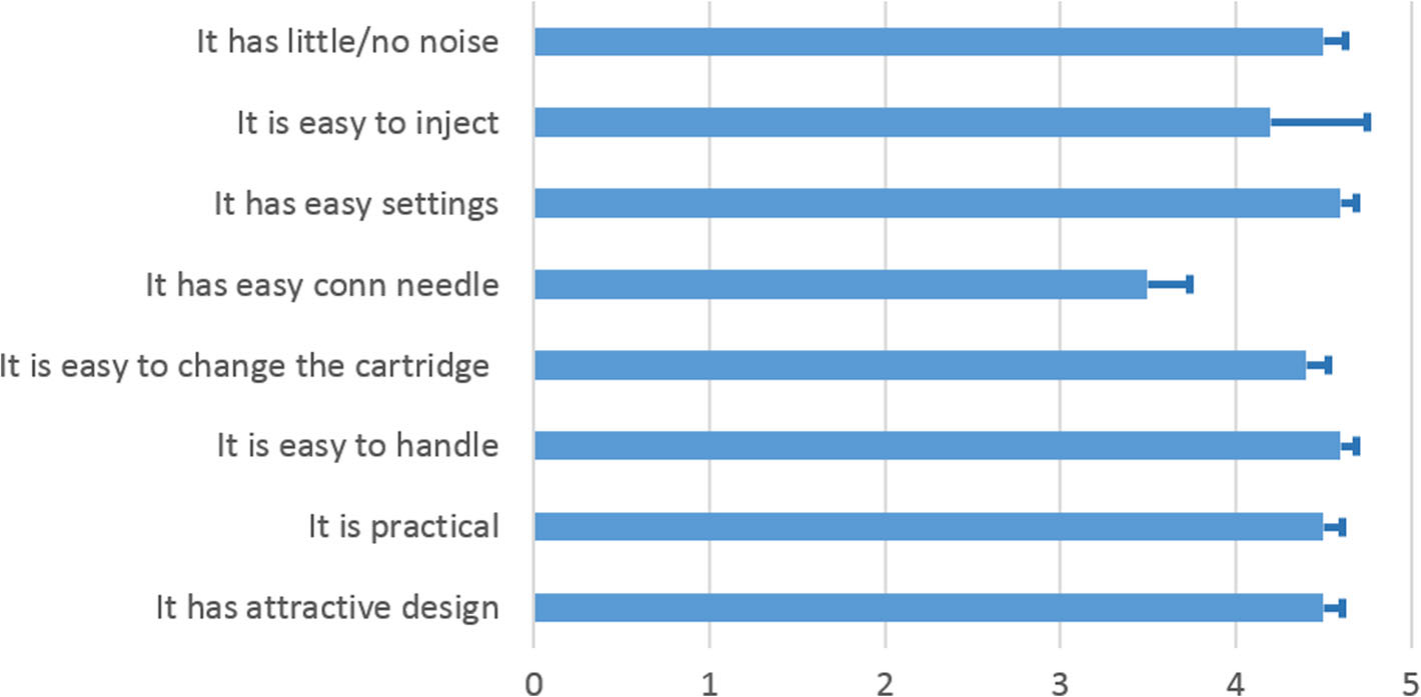
Responses to convenience questionnaire (*n* = 58). Mean scores and standard error bars are shown

Of the 64 patients in the FAS, 58 (90%) assessed RebiSmart®. Median score was 5 (highest) for all questionnaire items, while mean values are shown in Fig. 5. ED was documented for 19 patients (29% of the Safety Population). The most common reason was ‘patient’s decision to quit treatment’ (8/64, 12.5%), followed by ‘adverse event’ (4/64, 6.2%). Among the four cases where the drug was discontinued due to AEs, pregnancy was the reason in one, while in the other three cases the reasons were fatigue, malaise, anorexia, pyrexia and infections.

**Fig. 6:**
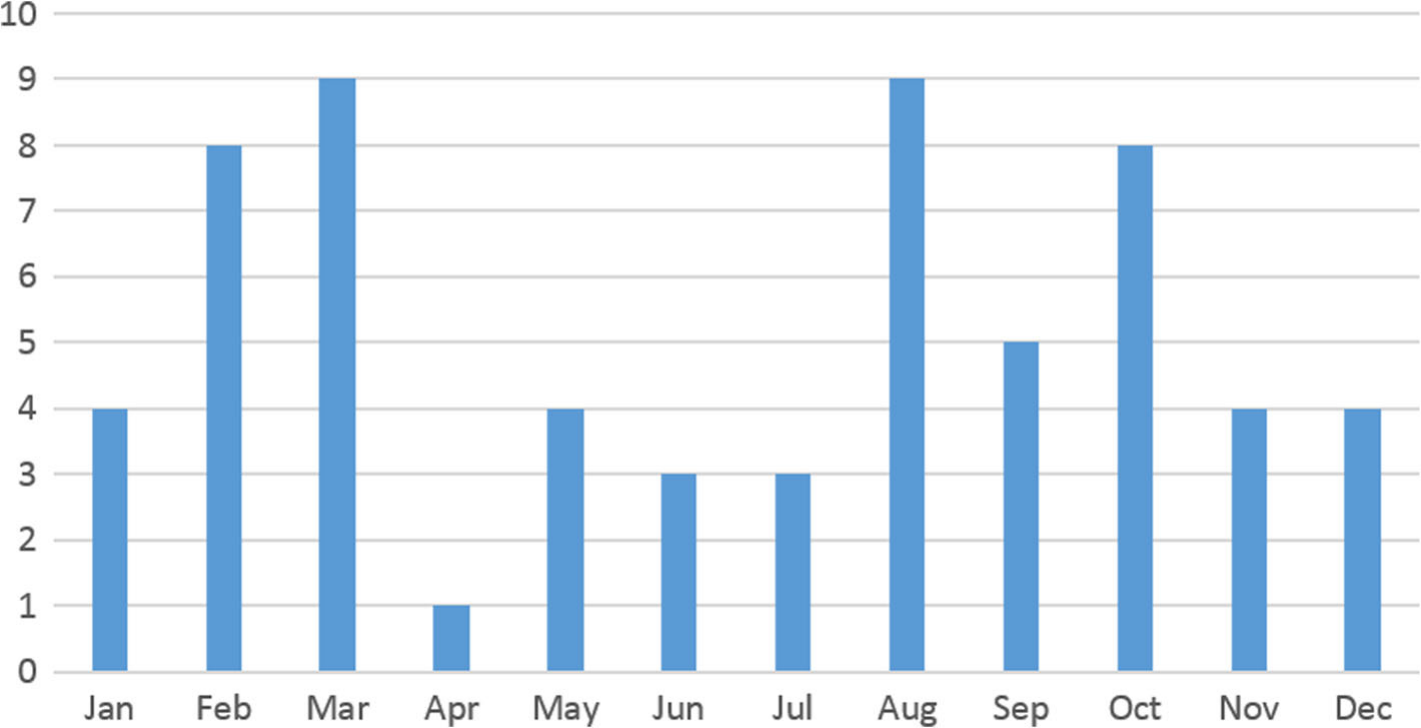
Monthly variation of reports of flu-like syndrome and of related symptoms (headache, malaise, myalgias) (*n* = 62)

Treatment with Rebif® using RebiSmart® was well tolerated. No new safety signals were detected through this study. Sixty two reports of flu-like syndrome and of related symptoms (headache, malaise, myalgia) were obtained. Figure 6 shows the monthly distribution of these reports. While the distribution is not even throughout the year (*p* < 0.001, χ^2^ test), peaks are observed in the spring, summer and autumn; this speaks against an effect of hot weather on the frequency and gravity of flu-like syndrome. Furthermore, monthly reports of flu-like syndrome did not correlate with monthly adherence (*r* = 0.14, *p* = 0.66).

RebiSmart® was evaluated by study participants as easy to use and convenient, giving an average score of 4.5 or above in most items of the convenience questionnaire (Fig. 5). The lower score (average 3.5 ± 1.79) was given to the item “it has easy connection needle”.

## Discussion

To our knowledge, this is the first prospective study to assess yearly, seasonal and monthly adherence to, and efficacy, safety, and tolerability of Rebif® for RRMS administered with an electronic autoinjector. In a previous study seasonal adherence to the interferons and glatiramer acetate had been studied retrospectively, by means of patient-administered questionnaire [7]. Here, adherence data was objectively captured by the autoinjector electronically and therefore not subject to patient reporting errors [2, 13].

In our study cumulative 12-month adherence to Rebif® was very high (97.93 ± 5.704, FAS), confirming the findings of a previous 12-month and of two 12-week user trials with the same autoinjector [2, 11, 14], (97.0 ± 7.3% cumulative 12-month adherence [2], 90.3% of patients with > 90% adherence [14], and 88.2% of patients having administered ≥80% of scheduled injections, with 67% administering all scheduled injections [11] respectively). The use of an intramuscular IFN b-1a autoinjector in another study resulted in monthly compliance rates of 87.5–96.2%, supporting the notion that an autoinjector may contribute to high compliance [15].

Interestingly, the COMPLIANCE study investigators [7], which took place in Spain, where weather conditions are similar to Greece’s, reported findings seemingly opposite to ours, namely that seasons had a considerable impact on adherence. The authors comment in the discussion that they found a correlation between summer months and non-adherence, however they acknowledge that this association was not statistically significant. Furthermore, 81% of their patients reported that seasons did not affect their adherence. Hence, the data in the COMPLIANCE study support our finding that seasons do not affect adherence.

Thirty one patients (48%) missed at least one injection during the study period. The main reasons for non-adherence (Table 5) are in agreement with previous reports [2, 5, 16]. RebiSmart® was evaluated by study participants as easy to use and convenient (Fig. 5). The lower score was given to the item “it has easy connection needle” and this might be an aspect of the device that can be improved.

Treatment with Rebif® was efficacious; 87% of the per-protocol population were relapse free at month 12, which compares favorably with the rates of 66.8% at 48 weeks and 53.3% at 96 weeks with the same serum-free Rebif® formulation administered manually or with a mechanical autoinjector [16, 17]. Mean number of relapses was significantly lower at month 12 compared to the pre-treatment year. These numbers are consistent with those recently reported for RebiSmart® [2], yet lower compared to the ARR obtained for Rebif® in a series of recent clinical trials where the latter was used a comparator (Rebif® vs Alemtuzumab in CARE-MS-I and CARE-MS-II where ARR for Rebif® was 0.39 ± 0.907 and 0.52 ± 1.01 respectively [18, 19] and Rebif® vs Ocrelizumab in OPERA-I and OPERA-II where ARR for Rebif® was 0.29 ± 0.72 and 0.29 ± 0.73 respectively [20]. The mean number of relapses in the 12 months pre-treatment was also higher in these studies: 1.33 ± 0.64 and 1.34 ± 0.73 in OPERA-I and II [20], 1.8 ± 0.8 and 1.5 ± 0.75 in CARE_MS I and II respectively [18, 19]. These differences in the study populations, as well as in the design of the trials, might account for the lower post-treatment relapse rate that we have observed. On the other hand, in the SMART trial, which recruited a similar patient population in terms of pre-treatment relapses, the one-year pre- and post- treatment ARR was comparable [2].

3-month confirmed disability progression at the end of the study period was observed in 10 patients (21%), while in 19 (40%) patients EDSS remained stable and in 18 (39%) it improved. Overall, in the PPS mean EDSS change of 0.17 ± 1.13 was not significant. It is notable that the change in EDSS from baseline was not related to the 12-month either pre- or post-treatment relapses (Fig. 3), while the mean number of relapses in the 12-month pre- and post- treatment period did not differ significantly among patients in whom EDSS had progressed, remained stable or improved at the end of the study (Fig. 4). Albeit there was a trend towards a higher mean number of relapses in the 12-month post-treatment period in those with EDSS progression, this difference did not reach statistical significance. It should be noted that these correlation analyses are post-hoc and should be treated with caution, as they are statistically under-powered.

Despite the relatively short observation period, these findings add to the on-going debate on the relation between relapses and disability in MS. Relapses and disability progression are two important clinical characteristics of MS. Relapses are the clinical expression of inflammatory insults localized at different parts of the central nervous system, whereas disability progression is the phenotypic expression of ongoing demyelination, axonal loss and gliosis [21]. In an earlier study of 1844 patients who had MS for 11 ± 10 years, it was found that once a certain clinical threshold is reached (namely, 4 on the EDSS), the progression of disability is not further affected by relapses. This, according to the authors, suggests that there is a dissociation between the pathophysiological mechanisms underpinning relapses and disability progression [21]. A more recent observational study of 162 MS patients treated with interferon beta for at least 2 years, found that compared to patients with no relapses in the first 2 years, those with 1 or ≥ 2 relapses were more likely to exhibit early sustained disability progression (Hazard Ratio for 1 relapse: 3.4, *p* = 0.05; Hazard Ratio for ≥2 relapses: 4.3, *p* < 0.001). However, there was no statistically significant difference between patients that had 1 or ≥ 2 relapses [22]. Finally, a real world evidence study comparing alemtuzumab, interferon beta, fingolimod, or natalizumab in terms of relapses and disability progression showed that despite the fact that alemtuzumab was associated with a lower ARR than Rebif® (0.19 [95% CI 0.14–0.23] vs 0.53 [0.46–0.61], *p* < 0.0001), it was associated with similar probabilities of both disability accumulation (hazard ratio 0.66 [95% CI 0.36–1.22], *p* = 0.37) and disability improvement as Rebif® (0.98 [0.65–1.49], *p* = 0.93) at 5 years [23]. Our results favor those studies that support a dissociation between relapses and disability progression, calling for more research on the pathophysiological mechanisms underpinning this phenomenon and on the appropriate treatment strategies.

A limitation of our study is its relatively small size. However, it is the only study that we are aware of, in which seasonal adherence to interferon treatment for MS is evaluated by means of an autoinjector, rather than by patient-administered questionnaires. This increases the objectivity of the measurements. Furthermore, the sample size for this study was calculated based on the primary endpoint, namely 12-month adherence, due to the lack of published data regarding the seasonal and monthly adherence. As, according to our findings, 12-month, seasonal and monthly adherence are similar and have higher mean values and lower standard deviations than what was assumed during sample size calculations, the recruited number of patients was adequate to also estimate seasonal and monthly adherence.

The very high adherence rate that we have observed could have been confounded by factors such as higher educational level, occupation or willingness to participate in a clinical study. While the latter is a common factor in all studies, our sample was balanced in terms of educational level (high school – university), social status (married or not) and occupation (public/private sector). Furthermore, we did not observe any discrepancies in the adherence rates, which was high in all these subgroups.

While the observational design of the study and its 12-month duration are suitable for evaluating adherence (primary endpoint), they are less relevant to the efficacy measurements (relapse rate and disability progression), which were however secondary endpoints and should be interpreted with caution. Nevertheless, the results that we have obtained on relapse rate are consistent with those published in the literature, while our finding that pre- and post-treatment relapses are not related to disability progression or improvement at 12-months adds to the on-going discussion on the matter.

## Conclusions

In conclusion, treatment with Rebif® using RebiSmart® was well tolerated and adherence exceeded 97% in a real world setting. There was no association of adherence with specific time periods of the year or geographical areas of Greece, which implies that weather conditions are not among its important determinants. Our data shows that Rebif® is effective in decreasing annual relapse rates, however there no correlation between ARR and disability progression.

## Abbreviations

ADR: Adverse Drug Reaction
AE: Adverse Event
ANOVA: Analysis of Variance
DMD: Disease Modifying Drug
ED: Early Discontinuation
EDSS: Expanded Disability Scale Score
FAS: Full Analysis Set
MS: Multiple Sclerosis
PPS: Per Protocol Set
RRMS: Relapsing Remitting Multiple Sclerosis
SADR: Serious Adverse Drug Reaction
SAE: Serious Adverse Event
scIFN-β1a: Subcutaneous Interferon β1a
SmPC: Summary of Product Characteristics
STROBE: STrengthening the Reporting of OBservational studies in Epidemiology
